# Altered Elemental Distribution in Male Rat Brain Tissue as a Predictor of Glioblastoma Multiforme Growth—Studies Using SR-XRF Microscopy

**DOI:** 10.3390/ijms23020703

**Published:** 2022-01-09

**Authors:** Karolina Planeta, Zuzanna Setkowicz, Mateusz Czyzycki, Natalia Janik-Olchawa, Damian Ryszawy, Krzysztof Janeczko, Rolf Simon, Tilo Baumbach, Joanna Chwiej

**Affiliations:** 1 Faculty of Physics and Applied Computer Science, AGH University of Science and Technology, Al. Mickiewicz 30, 30-059 Krakow, Poland; Karolina.Planeta@fis.agh.edu.pl (K.P.); Natalia.Janik-Olchawa@fis.agh.edu.pl (N.J.-O.); 2 Institute of Zoology and Biomedical Research, Jagiellonian University, Golebia 24, 31-007 Krakow, Poland; Zuzanna.Setkowicz-Janeczko@uj.edu.pl (Z.S.); K.Janeczko@uj.edu.pl (K.J.); 3 Laboratory for Applications of Synchrotron Radiation, Karlsruhe Institute of Technology, Kaiserstr. 12, D-76131 Karlsruhe, Germany; Czyzycki@kit.edu (M.C.); Tilo.Baumbach@kit.edu (T.B.); 4 Faculty of Biochemistry Biophysics and Biotechnology, Jagiellonian University, Golebia 24, 31-007 Krakow, Poland; Damian.Ryszawy@uj.edu.pl; 5 Institute for Photon Science and Synchrotron Radiation, Karlsruhe Institute of Technology, Hermann-von-Helmholtz-Platz 1, D-76344 Eggenstein-Leopoldshafen, Germany; R.Simon@kit.edu

**Keywords:** glioblastoma multiforme (GBM), animal models of GBM, multi-elemental analysis of rat brain, synchrotron X-ray fluorescence microscopy, U87mg, T98g

## Abstract

Glioblastoma multiforme (GBM) is a particularly malignant primary brain tumor. Despite enormous advances in the surgical treatment of cancer, radio- and chemotherapy, the average survival of patients suffering from this cancer does not usually exceed several months. For obvious ethical reasons, the search and testing of the new drugs and therapies of GBM cannot be carried out on humans, and for this purpose, animal models of the disease are most often used. However, to assess the efficacy and safety of the therapy basing on these models, a deep knowledge of the pathological changes associated with tumor development in the animal brain is necessary. Therefore, as part of our study, the synchrotron radiation-based X-ray fluorescence microscopy was applied for multi-elemental micro-imaging of the rat brain in which glioblastoma develops. Elemental changes occurring in animals after the implantation of two human glioma cell lines as well as the cells taken directly from a patient suffering from GBM were compared. Both the extent and intensity of elemental changes strongly correlated with the regions of glioma growth. The obtained results showed that the observation of elemental anomalies accompanying tumor development within an animal’s brain might facilitate our understanding of the pathogenesis and progress of GBM and also determine potential biomarkers of its extension. The tumors appearing in a rat’s brain were characterized by an increased accumulation of Fe and Se, whilst the tissue directly surrounding the tumor presented a higher accumulation of Cu. Furthermore, the results of the study allow us to consider Se as a potential elemental marker of GBM progression.

## 1. Introduction

Animal models of diseases have a great importance in experimental studies, which cannot be performed on humans for ethical reasons. They improve the knowledge about both physiological and pathological processes taking place in the organisms. Animal models are commonly used for the study of disorders of different origins and also developing/testing new therapeutic substances or strategies.

Tumors of the central nervous system are the 10th most common cause of death among both men and women. Gliomas, originating from glial cells responsible for neurons support, constitute about 33% of all brain tumors. Depending on the cells’ morphology, their proliferation ability and aggressiveness, four grades of malignancy are distinguished for gliomas. The group of gliomas with the highest degree of malignancy includes glioblastoma multiforme (GBM). GBM is a rapidly progressive and infiltrative brain tumor, characterized by an intensive formation of new blood vessels and the presence of necrotic and hemorrhagic foci within its structure. It accounts for 16% of all brain tumors and stands out by high mortality—the median survival of patients from diagnosis is about several months [1,2].

Many interdisciplinary research groups have been currently conducting investigations aiming at gaining more comprehensive knowledge about gliomas etiology and developing new and effective methods of its treatment. Some of the studies concerning the pathogenesis and the progress of GBM are conducted on tissue samples taken from humans suffering from GBM. In these cases, however, one should remember that the obtained results may be influenced by many factors including the applied therapy [3,4]. In turn, due to ethical considerations, the testing of new antitumor substances/drugs cannot be carried out on humans and therefore animal models, usually based on rodents, are used for both purposes [5,6,7].

Testing new therapies of GBM based on animal models of the disease requires a deep knowledge about the morphological and biomolecular changes introduced in the brain through the developing tumor. In this study, we compared topographic and quantitative elemental anomalies appearing in the brain as a result of the implantation of three different types of GBM cells. For this purpose, Wistar rats were subjected to the intracranial implantation of human GBM cell lines U87mg and T98g as well as patient-derived glioma cells. Then, we applied synchrotron radiation-based X-ray fluorescence (SR-XRF) spectrometry for the elemental mapping of brain tissue slices taken from the area of tumor implantation. SR-XRF is a valuable tool allowing for the investigation of changes in both the distribution and accumulation of elements. This sensitive and non-destructive method of multi-elemental analysis offers low detection limits and a short acquisition time. The above-mentioned properties enable the use of the analytical technique to investigate the elemental topography of biomedical samples [8,9,10,11]. As a result of the SR-XRF measurements, we obtained two-dimensional maps of P, S, K, Ca, Fe, Cu, Zn and Se distribution. In addition, we compared them with the corresponding microscopic images of the examined tissues in order to identify local anomalies specific for the animals implanted with particular GBM cells.

Each of the analyzed elements is important for the purposes of our study, in terms of their involvement in both the physiological as well as pathological processes occurring in the cells. Phosphorus is a macronutrient playing an important role in the cells’ metabolism and constituting a building component of cell membranes. Sulfur compounds may increase the sensitivity of GBM cells to radiation therapy [12]. In turn, potassium channels are observed to be abnormally expressed in glioma cells [13]. The occurrence of calcium deposits is a feature accompanying intracranial tumors with a different grade of malignancy [14]. Copper is consider as a cofactor involved in the process of angiogenesis [15]. Iron and zinc are the elements essential for the proper regulation of cells’ growth and DNA synthesis [16,17]. Furthermore, an increased iron uptake was observed for brain tumor cells [18]. Whilst selenium is crucial for the proper functioning of immune system cells and it is also involved in the processes of cell proliferation and apoptosis [19].

This is our second study concerning the elemental abnormalities of a rat’s brain following the implantation of glioma cells. The first one, performed with the use of total reflection X-ray fluorescence (TXRF), provided us with information about tumor-induced changes in elemental concentrations averaged over the brain hemispheres. The obtained results allowed us to classify the U87mg cell line as the most aggressive among the investigated GBM cells and to identify the elements potentially involved in tumor progression [20]. As the distribution of the elements in the analyzed tissues may not be homogenous, in the current study, we focused on the local elemental anomalies of the brain tissue and particular attention was directed toward the differences between the region of the tumor and its surrounding. This approach enabled us to obtain more precise information on the contribution of the analyzed elements to the process of GBM pathogenesis and progress.

## 2. Results

### 2.1. Outline of the Experiment

Five groups of male Wistar rats were the subject of the study. Three of them were intracranially implanted with human GBM cells of different origin (U87mg and T98g cell lines and patient-derived glioma cells) suspended in the culture medium. Two groups constituted the controls. One consisted of animals implanted with cells growth medium, whilst the second included naive normal rats. Three weeks after the procedure of implantation, the animals were sacrificed, and brain sections from the area of implantation were taken and prepared for measurements. Detailed descriptions of the experiment assumptions as well as the measurement conditions are included in the Materials and Methods section.

### 2.2. Data Analysis

As a result of the raster scanning of tissue slices with the X-ray beam, a set of XRF spectra, together with the information about the position of the beam for which they were recorded, were obtained for each sample. Spectral analysis was performed with the PyMca software ver. 5.0.2 [21], which enabled us to gather information about the net peak areas of the *Kα* lines for the elements under interest and calculate the areal densities of the elements ($M_{T}$) for the examined tissue points in accordance with Equation (1).

(1) $$M_{T}=\frac{Y_{T}}{S\times Y_{T}^{N}},$$

$M_{T}$—the areal density of the analyzed element in the tissue sample (μg/cm^2^);
$Y_{T}$—the net peak area of the *Kα* line of the measured element for the tissue sample (a.u.);
*S*—sensitivity for the measured element (cm^2^/μg);
$Y_{T}^{N}$—the incoming X-ray beam normalization factor for the tissue sample (a.u.).

The sensitivities *S* for the measured elements were determined in the calibration process and, for this purpose, the MICROMATTER XRF calibration standards (GaP, KCl, CaF_2_, Ti, Fe, Cu, ZnTe, Se, CsBr, RbI, SrF_2_) were used. Sensitivity values were quantified according to Equation (2)

(2) $$S=\frac{Y_{S}}{M_{S}\times Y_{S}^{N}},$$

$Y_{S}$—the net peak area of the *Kα* line of the measured element for the standard sample (a.u);
$M_{S}$—the areal density of the analyzed element in the standard sample (μg/cm^2^);
$Y_{S}^{N}$—the incoming X-ray beam normalization factor for the standard sample (a.u.).

**Figure 1:**
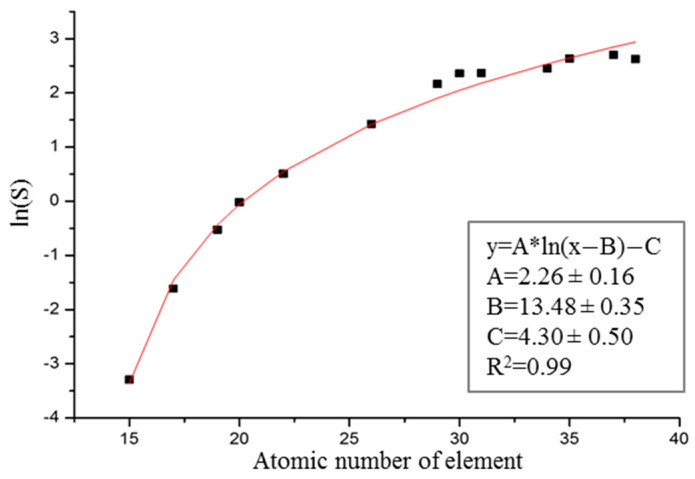
Sensitivity curve determined from the measurements of the MICROMATTER XRF calibration standards.

The sensitivities *S* were then used to determine the calibration curve that is presented in Figure 1.

### 2.3. Limits of Detection

The limit of detection $LOD_{ij}$ (μg/cm^2^) for the element *i* in the tissue point *j* was calculated in accordance with Equation (3)

(3) $$LOD_{ij}=3.29\times \frac{\sqrt{Y_{Bij}}}{Y_{Nij}}\times M_{Tij}$$

$Y_{Bij}$—the integrated area of the background under the *Kα* line of element *i* in the spectrum recorded for tissue point *j* (a.u.);
$Y_{Nij}$—the net peak area of the *Kα* line of element *i* in the spectrum recorded for tissue point *j* (a.u.);
$M_{Tij}$—the areal density of element *i* in tissue point *j* (μg/cm^2^).

To estimate the detection limit of the element *i* for the brain tissue ($LOD_{i}$), the results obtained for the samples taken from normal rats were used. From each tissue slice, we randomly selected 30 points localized in the striatum region in the right hemisphere, calculated $LOD_{ij}$ for each point, which was followed by averaging the results from all the points and for all the animals. The uncertainties of the determined $LOD_{i}$ values were calculated as the standard deviation of the mean value, according to Equation (4).

(4) $$SD_{LODi}=\sqrt{\frac{\sum_{1}^{n}{\left(LOD_{ij}-LOD_{i}\right)}^{2}}{n\times (n-1)}}$$

$LOD_{i}$—the detection limit of element *i* for the brain tissue, calculated as an average of the results for all the selected tissue points (μg/cm^2^);
*n*—the number of examined points.

The final values of such calculated detection limits of elements, together with their uncertainties, are presented in Table 1.

### 2.4. Morphological Evaluation

**Figure 2:**
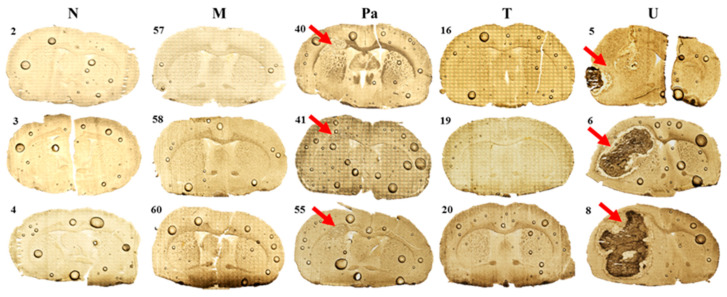
Microscopic images of tissue slices taken from the place of the implantation from 3 representative animals of each examined experimental group (N—normal rats; M—animals receiving DMEM; Pa, T and U—animals implanted with GBM cells taken from patient and T98g or U87mg cell lines, respectively). Red arrows indicate tumor bulk developed after the implantation of glioma cells.

A morphological evaluation of the tissues taken from the place of the implantation yielded the development grade of the tumors that originated from the used GBM cell lines. Examples of the microscopic images obtained for the representative samples from the five examined animal groups are presented in Figure 2. It can be seen that the implantation of both U87mg cells and patient-derived cells led to the development of a tumor within the affected rat’s brain hemisphere. In the case of U87mg cells, a massive tumor, often involving the entire hemisphere, was observed. After implanting T98g cells, no signs of tumor development were found. However, in the regions where the glioma cells were implanted, similarly to the case of DMEM administration, morphological changes indicating nervous tissue damage were found.

### 2.5. Qualitative Elemental Analysis

**Figure 3:**
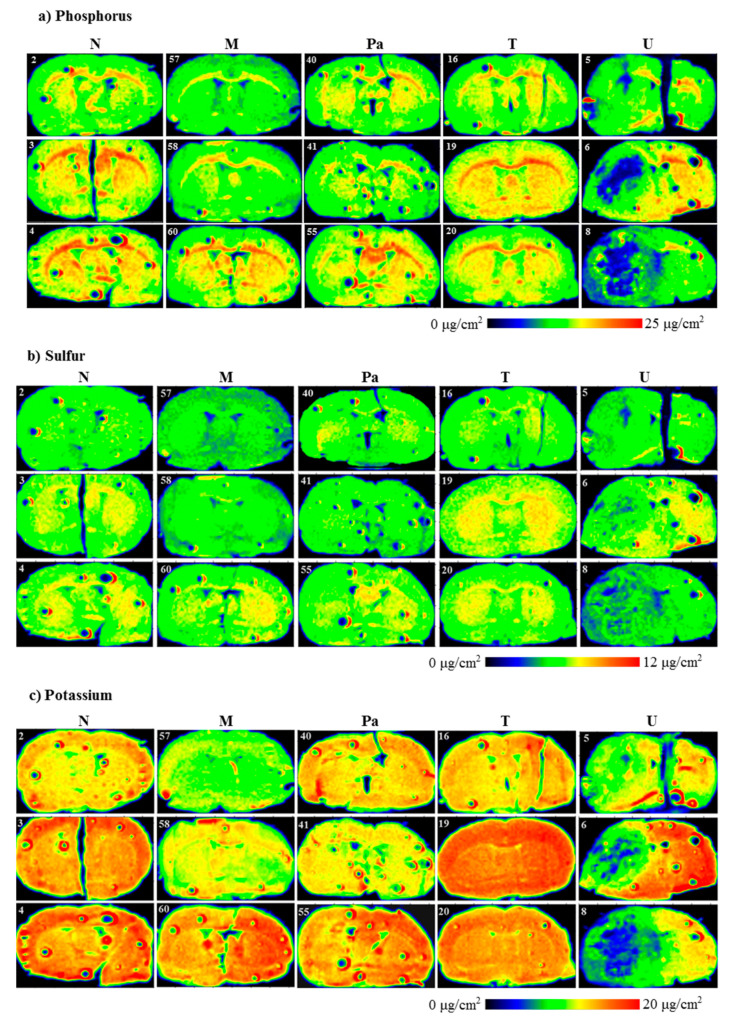

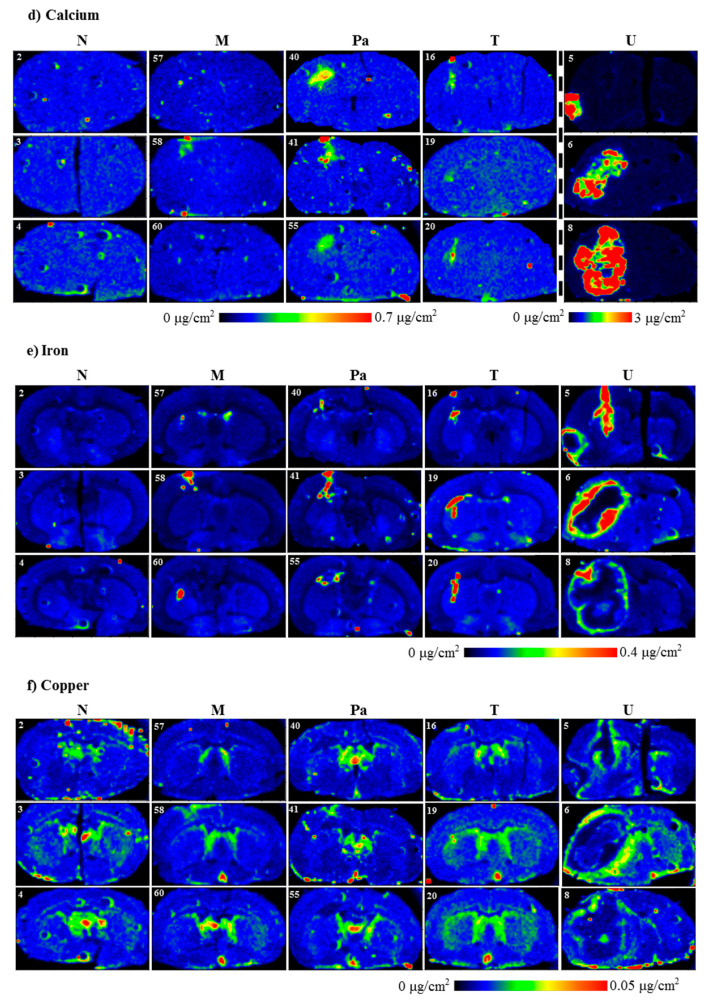

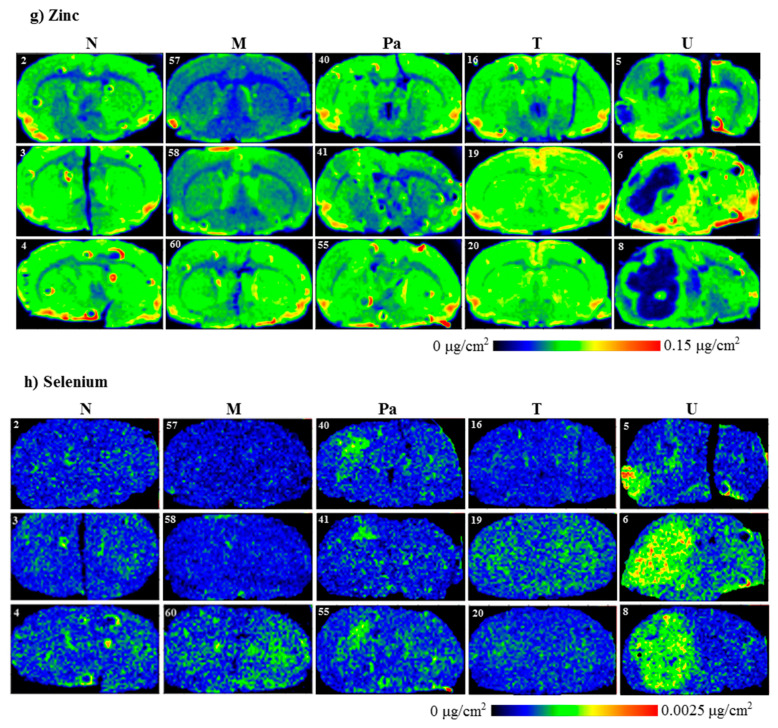
Topographic elemental maps of brain slices taken from the rats of all experimental groups for: (**a**) phosphorus, (**b**) sulfur, (**c**) potassium, (**d**) calcium, (**e**) iron, (**f**) copper, (**g**) zinc and (**h**) selenium. Color scales express areal densities of elements in μg/cm^2^. In the case of Ca, large differences in areal densities were observed between groups N, M, Pa, T and group U. Two different scales were applied for better visualization of the distribution of this element.

Evaluating the distributions of the elements under interest (P, S, K, Ca, Fe, Cu, Zn and Se) in the scanned brain slices was the first step of our investigation. The results of the topographic elemental analysis for the representative samples that originated from all the experimental groups are provided in Figure 3.

As one can see from Figure 3a, a clear decrease in P accumulation in the implanted brain hemisphere and especially in the area of tumor development was found for the rats from the U group. A reduced element content was noted compared to the naive hemisphere within the same sample, as well as compared to the left hemisphere of the normal animals. A similar relation was observed for the animals implanted with patient-derived glioma cells. Additionally, for them, the area of the tumor determined from microscopic examinations corresponded with the region of a slight decrease in phosphorus accumulation. In the case of groups M and T, we did not observe any changes in P distribution in the regions of interest.

Topographic analysis of the maps of S distribution (Figure 3b) illustrated changes in the accumulation of this element in the left hemispheres of the animals implanted with the U87mg cells. For this group of rats, the areas of the reduced S content, compared to the intact hemisphere as well as to the left hemispheres taken from the control rats, were observed. These differences were not found in the case of the remaining experimental groups. A similar observation was made in the case of K distributions. The clear decrease in its accumulation occurred only in the areas corresponding to the tumors developed in the brains of the animals from the U group.

Ca and Fe are two elements, the distribution of which was disturbed both within the place where the glioma cells were implanted and culture medium was administered. The accumulation of both elements increased in the regions of DMEM administration and T98g cells injection (M and T groups, respectively), as well as the tumor development in the case of the Pa and U groups.

The topographic maps recorded for Ca and Fe showed a great heterogeneity of element accumulation in the tumor bulk developed from U87mg cells. A significant increase in Ca content was found in the inner area of the tumor, while Fe accumulated more in its border layers. Furthermore, regions of an elevated accumulation of Ca and Fe corresponded with the tumor areas presenting a different morphological structure, which can be seen in Figure 2, illustrating microscopic images of the scanned tissue sections.

The results obtained for Cu showed a substantially elevated areal density of this element in the tissue directly adjacent to the tumor developed from the U87mg cells. Additionally, in the case of the U group, the distribution of Cu within the tumor was heterogeneous and its level was lower in the external layer than the inner region. In turn, the level of Cu was generally lower in the tumor compared to its surroundings for the samples taken from the animals belonging to the Pa group.

Zn accumulation showed anomalies in the samples taken from the animals representing the Pa and U groups. A decrease in this element content was recorded in the area of the tumor developed from the U87mg cells. Furthermore, the reduced Zn level occurred mainly in the inner part of the tumor and was usually correlated with the area of lower P accumulation. The tumor developed from patient-derived cells presented the heterogeneous Zn distribution with a slightly elevated content compared to the surroundings.

In the examined tissue slices, Se was selectively accumulated in the tumors developed in the animals from the Pa and U groups. Moreover, its distribution within the tumor bulk was generally homogenous and corresponded with the area of the tumor determined based on microscopic images.

### 2.6. Quantitative Elemental Analysis

The second step of our study consisted of performing quantitative elemental analysis for the examined brain samples. Its main purpose was to evaluate the statistical significance of the differences in elemental accumulation in the places where T98g cells or DMEM were administered and their surroundings as well as the tumors developed from implanting either patient cells or U87mg cells and their surroundings. In this investigation, the samples representing the M, T, Pa and U groups were taken into account.

**Figure 4:**
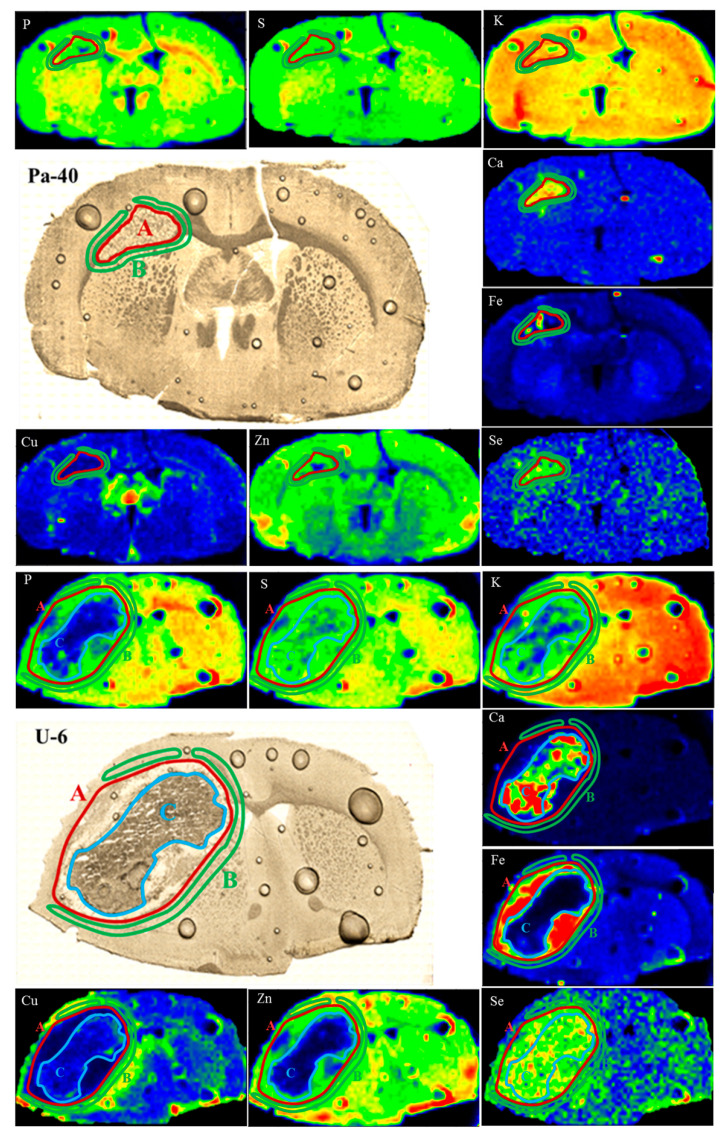
Regions of tumor (**A**), surrounding tissue (**B**) and tumor debris (**C**) marked in the maps of elemental distribution as well as microscopic images of brain slices taken from selected animals representing Pa and U groups.

First, based on the microscopic images of the tissues for the Pa and U groups, the areas corresponding to the place of tumor development were defined. When choosing the areas of interest for the samples originating from the M and T groups, we based the selections on the regions with the increased Fe accumulation that correlated with the places where T98g cells or DMEM were administered. For the brain samples taken from the rats representing the M, T and Pa groups, we defined two regions: first corresponding to the places where the medium or glioma cells were administered and second constituting their surroundings. In the case of the tumors developed from U87mg cells, due to the clear heterogeneity within their structure, we also defined an additional region inside the tumor bulk, which is further referred to as the tumor debris. The mentioned areas, marked in the elemental maps and microscopic images of the selected samples representing the Pa and U groups, are presented in Figure 4.

**Figure 5:**
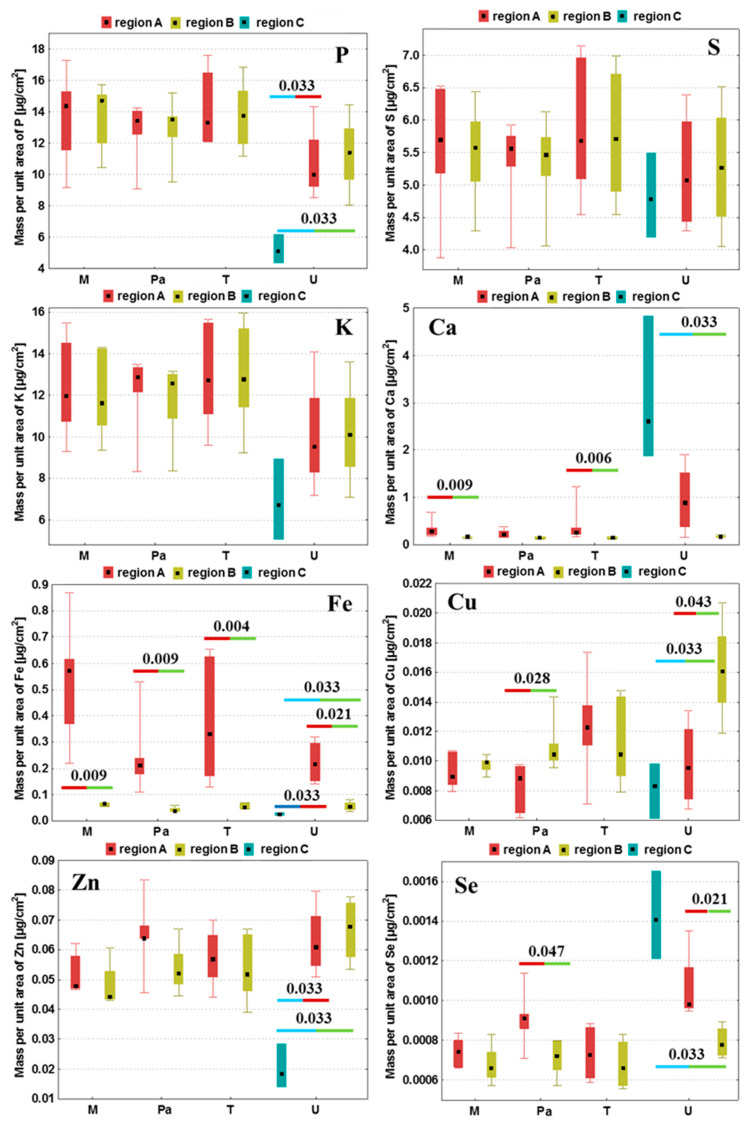
Medians, minimal and maximal values as well as interquartile spans of areal densities of P, S, K, Ca, Fe, Cu, Zn and Se in the examined tissue regions obtained for groups M, Pa, T and U. Region A—tumor (groups Pa and U) or place where DMEM/T98g cells were administered (groups M and T, respectively); region B—surroundings of the tumor or place where DMEM/T98g cells were administered; C—tumor debris defined in the case of the U group. Statistically significant differences between the compared areas within the same experimental group were marked with *p*-values of the *U* test.

For each examined brain slice, the mean quantities of P, S, K, Ca, Fe, Cu, Zn and Se were calculated in the regions mentioned before. Then, based on the results obtained for the whole experimental group, the median, minimal and maximal values of areal densities of the analyzed elements were determined and the obtained results were presented in the form of box-and-whiskers plots in Figure 5. We applied the non-parametric Mann–Whitney *U* test at the confidence level of 95% [22] to evaluate the statistical significance of the differences in the elemental composition between the compared regions within a given animal group. All the statistically relevant differences were determined based on this test and are marked with their *p*-values in Figure 5.

As one can see from Figure 5, the tumors developed after the implantation of U87mg cells presented numerous statistically significant differences in the elemental distribution, both comparing to their surrounding and within the tumor bulk, which resulted from their heterogeneous structure. Defined for the U group, tumor debris was characterized by the diminished quantities of P, Fe and Zn compared both to the tumor as well as to the tissue adjacent tumor. Furthermore, in this region, we observed a reduced accumulation of Cu and an increased content of Ca and Se, in relation to the surrounding tissues.

The comparison of tumors developing in the animals representing the Pa and U groups showed a similar pattern of elemental differences between the tumor and its surroundings. The tumors developed from both U87mg cells and from patient-derived cells were characterized by a higher accumulation of Fe and Se compared to the adjacent tissues. In turn, the tumor’s surroundings revealed an elevated content of Cu in relation to its bulk. Additionally, the M and T groups were similar one to the other in terms of the observed elemental anomalies. The places where the T98g cells or DMEM were administered presented an elevated Ca and Fe accumulation compared to the surrounding tissue.

Fe accumulation changes at the place of administration were common for the samples taken from all the experimental groups and the amount of this element was always higher there in relation to the surroundings.

## 3. Discussion

A significant number of publications cover the involvement of trace and major elements in the process of carcinogenesis. The differences in elemental accumulation between healthy and neoplastic tissues are observed in the case of various types of tumors [23,24,25,26]. They may result, among others, from an increased demand for some elements during the tumor development, which may, in turn, be associated with the changed metabolism of tumor cells or their higher proliferation ability [27,28,29,30]. With knowledge of the elemental abnormalities that are characteristic for a particular neoplasm, some elements or their inhibitors might be selected and then tested as potential agents supporting the treatment against the tumor [31,32,33].

The subject of our study was glioblastoma multiforme, one of the most aggressive brain tumors, characterized by very high mortality among patients. We selected two human GBM cell lines—U87mg and T98g—and implanted them into the left cerebral hemispheres of rats. Cells derived from a patient suffering from GBM were used in an identical manner. Our investigation presented in this study is the continuation of our previous study, where the TXRF method was applied to determine the mean concentrations of P, S, K, Ca, Fe, Cu, Zn and Se in brain hemispheres taken from rats that were implanted with the same glioma cells [20]. The results of the above-mentioned study provided us with a general view of the elemental changes caused by the tumor development and allowed us to associate the revealed anomalies with the degree of aggressiveness of the selected GBM cells. As TXRF spectrometry only enables a bulk analysis of the examined samples, there was still an open question about the local elemental anomalies within the GBM-affected brain tissues, especially within the tumor bulk and its surroundings. Therefore, we applied SR-XRF microscopy to perform the topographic and quantitative elemental analysis of the brain slices taken from the area where the glioma cells were implanted. Data concerning the distribution and accumulation of the examined elements were correlated with the information about the tissue histology. The quantitative analysis followed by statistical evaluation allowed us to determine the elements for which accumulation differs significantly between the tumor area and its surroundings. In the discussion presented below, based on evidence in the available literature, we try to indicate the possible causes of the observed elemental anomalies.

Based on histological examinations, Martin and Lemmen distinguished four calcification patterns of intracranial tumors. They classified benign lesions to groups that frequently show a tendency to calcification. In the case of high-grade gliomas and glioblastomas, calcifications were rarely seen [14]. However, cases of glioblastomas with the presence of calcified regions were also reported [34,35,36,37,38]. In the literature, one can find reports on clinical cases, where imaging examinations performed few years before brain tumor diagnosis revealed calcifications in the area of cancer development [39,40]. Mallya et al. and Kroh et al. suggested that the presence of calcified regions may be associated with a longer survival period [36,37]. Bähr et al. analyzed the cases of patients suffering from GBM who had undergone therapy with Bevacizumab. The authors observed the occurrence of calcifications in gliomas after the treatment. Moreover, the median survival of patients in which tumor calcification occurred was higher than in those without such changes [41]. Similar observations were made by Blumenthal et al. [42]. These results appear to be in agreement with the in vitro investigation, revealing that Bevacizumab induces a specific death of endothelial cells connected with massive Ca accumulation [43]. Halpin and Kingsley described two clinical cases of patients with an intracranial tumor for which calcifications’ disappearance was found. It was suggested that such a phenomenon might be a prognostic factor of high-grade tumor development and is probably associated with the progressive changes of tissue pH resulting from its growth [44].

In biological tissues, calcification foci usually state hydroxyapatite deposits composed of Ca and P. Our study showed, however, that the areas of increased Ca accumulation are usually correlated with the regions of diminished P amounts. This is especially visible for the tumor debris found in the U group. Such an observation is in agreement with the findings of Guisan et al., who also found the negative correlation between Ca and P quantities for brain tumor bulks [18]. We hypothesize that the alterations in Ca accumulation within the examined regions, revealed in our study, might be rather associated with the process of glutamate excitotoxicity. Glutamate is an excitatory neurotransmitter, whose concentration is normally regulated through the astrocytes’ uptake. Ye and Sontheimer showed that GBM cells are characterized by the diminished absorption of glutamate compared to the normal astrocytes. Furthermore, they release considerable amounts of this neurotransmitter to the extracellular environment. According to the authors, glioma cells may be responsible for the death of surrounding neurons due to this excessive glutamate release [45]. Under the conditions of the increased glutamate in the cells’ surroundings, glutamate-sensitive receptors are stimulated. Some of these receptors are permeable for Ca ions and their activation causes an elevated influx of this element into the cells. This, in turn, results in the activation of enzymes involved in the cell death and triggers a cascade of damaging processes, including the release of the reactive oxygen species, ATP depletion and membranes oxidation [45,46]. Clearly elevated Ca quantities, as well as inhomogeneity in its distribution, are observed within the tumor bulk developed from U87mg cells. This effect is mainly noticed in the region described as the tumor debris. Tumor debris probably constitutes a necrotic tissue, the presence of which is both one of the characteristic morphological features of GBM and a poor prognostic factor [47]. The decrease in the phosphorus content in the necrotic area may be explained by the depletion of ATP resources and the damage of cell membranes. Phosphorus is present in phospholipids’ building membranes and, therefore, their disintegration may be responsible for the reduced content of the element compared to a healthy tissue.

The topographic analysis of the brain slices taken from the rats subjected to the implantation of patient-derived cells also showed an increased deposition of Ca within the tumor. However, the element distribution was quite homogenous, which probably results from the fact that, in this case, the debris area was not formed within the tumor bulk. This, in turn, probably results from the differences in the aggressiveness of implanted GBM cells, as the presence of necrotic regions usually indicates a higher degree of malignancy and progression ability.

Glutamate excitotoxicity and the resulting elevated Ca accumulation are the common outcomes of the nervous tissue injuries occurring following trauma or cerebral ischemia [48,49,50]. The procedures performed in our study, necessary for implanting glioma cells, might lead to nervous tissue damage. This finding is confirmed by an increased Ca accumulation in the place where the culture medium was administered. Probably, the same effect is responsible for the significant increase in Ca observed within the area where the T98g cells were implanted.

Fe is a key micronutrient essential for cells’ growth and division. It is part of many proteins that enable proper cell functioning and are involved in processes such as oxygen transport, metabolism and DNA synthesis. On the other hand, Fe may also be potentially toxic for living cells by mediating free radicals’ formation. According to epidemiological reports, an elevated body content of Fe is associated with a higher cancer risk [16]. Guisun et al. found that the amount of Fe increases in brain tumors together with their malignancy [18]. Furthermore, it was shown that the expression of ferritin, the protein storing Fe within living cells, grows with the glioma grade. Transferrin is responsible for Fe transport in the blood plasma and its delivery to body cells [28]. Cancer cells of various origins, compared to healthy cells, are characterized with a overexpression of transferrin receptor 1 (TfR1), which is responsible for binding the Fe from the blood and, therefore, their uptake of Fe is higher [51]. In addition, Fe-chelators suppress the progression of tumors both in vitro and in vivo, what may indicate the significant role of the element in the process of carcinogenesis [52]. It was shown that in the case of gliomas, the overexpression of transferrin receptors (TfR) is associated with an enhanced tumor cell proliferation and neuronal death. An increased or decreased TfR expression results in the reduction in or acceleration of glioma progression, respectively. Furthermore, by promoting the release of glutamate, TfR stimulate the reduction in the neurons’ mass and provide a space for glioma development [53]. The importance of Fe for the metabolism of gliomas may be proven by the fact that 68-Ga, binding similarly to Fe by TfR, was successfully applied for the imaging of these tumors with positron emission tomography [54]. Furthermore, it was shown that elevated Fe uptake is observed for GBM stem-like cells being the self-renewing population of glioma cells involved in the tumor progression [28]. Stem-like cells may be located within the glioma core (in peri-vascular and peri-necrotic niches) or in its periphery, where they are involved in tumor invasion [55]. In our study, we observed the increased accumulation of Fe within the place of administration for all the experimental groups. The elevated Fe deposition within tumors developed from the patient-derived and U87mg cells may point at the regions of intensified proliferation having invasive potential. Especially for the tumors developed in the rats from the U group, the peripherally localized areas of higher Fe accumulation may be associated with the presence of the invasion-supporting GBM stem-like cells. In the case of the animals subjected to an intracerebral DMEM injection but also in the rats implanted with T98g cells and for which a tumor did not develop, a higher Fe content may result from tissue damage as a result of needle insertion and/or the subsequent hemorrhage.

The region of tumor debris, observed for gliomas developed from U87mg cells, is characterized by a significantly diminished accumulation of Zn, which is an element crucial for the proper functioning of living cells and takes part in the regulation of cells’ growth and division as well as DNA synthesis [17]. As previously mentioned, this region probably corresponds with the necrotic tissue where zinc-involving processes may just not occur and, because of this, the amount of the element may be lower there both compared to the tumor bulk and its surroundings.

Many studies indicate the involvement of Cu in the formation of new blood vessels. This process, referred to as angiogenesis, is crucial for the tumor development characterized by increased blood supply. Cu activates various proangiogenic factors and stimulates the proliferation of endothelial cells, which line the inner surfaces of vessels [15]. It was shown that the use of Cu chelators as well as the inhibition of its intracellular transport may suppress the process of angiogenesis in different types of tumors [56,57] and, therefore, clinical trials are conducted to verify if low-copper diets may improve the outcome of the anti-cancer treatment of patients [58]. An increased amount of Cu was found in the serum of patients suffering from neoplasms of various origins [59]. Similar observations were made in the case of patients diagnosed with inflammatory diseases as well as in animal models of these disorders. A higher concentration of copper in serum is probably associated with the increased level of ceruloplasmin—an inflammatory protein transporting Cu to tissues [60].

The results of our study showed a specific non-homogenous distribution of Cu within the tumor-affected hemisphere. The highest accumulation of the element was observed in the peri-tumoral area, and this effect was most pronounced for the U group. Such a result may point at the infiltrative character of the tumor and/or the ongoing angiogenesis related with its progression. Similar observations concerning the increased amount of Cu in the region surrounding human GBM were made by Dehnhardt et al. The authors suggested that the elevated quantity of the element in the peri-tumoral zone may result from the neovascularization as well as from inflammatory processes caused by the tumor development [61]. In turn, the decreased Cu accumulation observed in our study within the tumor bulk and debris probably resulted from the limited vascularization due to the neurodegenerative processes ongoing there.

Se is a well-known micronutrient involved in immunity stimulation and cancer prevention. Some of the Se-containing proteins protect against the damages induced by reactive oxygen species (ROS) considered as the inflammation and mutagenic factors leading to the carcinogenesis. Furthermore, through the cell cycle modulations, Se is involved in the repair of DNA and the apoptosis process [19]. Harmanci et al. examined the influence of selenomethionine on GBM cells and found that at low doses it promotes cell proliferation, while its high amounts stimulate cellular death [62]. An elevated accumulation of Se, compared to the healthy brain tissue, was found in various brain tumors of both human and animal origin [18,63,64,65]. Additionally, an increased accumulation of Se in the bulk of human GBM was usually accompanied by its lower concentration within the serum, which may prove the greater demand of neoplastic cells for this element [63]. All these reports are in agreement with our results, pointing at a significantly higher accumulation of selenium within the tumor bulk compared to the surrounding tissues. It should be also noted that Se is the only element whose distribution is quite homogenous within the tumor developed from U87mg cells. The Se quantity is elevated both in the tumor as well as in the debris area. In addition, the increased accumulation of the element clearly distinguishes the tumor bulks developed for the U and Pa groups from the areas where DMEM or T98g cells were administered when tumor development and expansion were not observed. Furthermore, among all the analyzed elements, only the region of increased Se quantity coincides with the extent of the neoplastic area, determined based on histological examinations. All these observations allow us to consider selenium as a potential elemental marker of tumor progression. We also suppose that the elevated accumulation of Se within a glioma bulk may result from an increased demand of proliferating tumor cells for this element and/or it may be a result of the immune system response to the ongoing tumor expansion and inflammation.

## 4. Materials and Methods

Detailed information concerning the used GBM cell lines, laboratory animals, pharmaceuticals and conditions of the experiment, including the procedure of implantation, are reported elsewhere [20]. Here, the most important assumptions of the performed research were briefly presented.

We used the three types of human glioma cells for the purposes of our investigation. These were two commercially available GBM cell lines (T98g and U87mg, both purchased from ATCC company) and the cells extracted directly from the tumor of a patient diagnosed with GBM. Isolation procedure of the patient cells was approved by the Bioethics Commission for the use of cellular material collected from patients in neurooncological operations (Decision no. 535/2017 of 13 June 2017, issued by the Bioethical Commission at the University of Nicolaus Copernicus in Toruń, Poland).

The subjects of the study were five groups of male Wistar rats at 9 weeks of age and each group consisted of six animals. Detailed characteristics of the experimental groups are presented in Table 2. Animal experiments were approved by the 2nd Local Institutional Animal Care and Use Committee (agreement no. 119/2016) and were performed with international standards.

Gender dependence in GBM is observed both in case of the occurrence and the survival rates, and men are more at risk that women rting GBM stem-like cells. p. uld be addressed [66]. An ideal animal model of GBM should involve both male and female individuals. Moreover, taking into account the variability of the sex hormones during the female life, such a study should include groups of animals at different developmental stages. Due to practical reasons and restrictions on the number of animals involved in the experiment, only male rats were used in this study. Nonetheless, due to the mentioned gender differences for GBM, we admit that similar research including female rats is needed and we plan to perform it in the future.

The procedure of implanting cells or administering DMEM into rat brains consisted of a few steps. The animals were pre-anesthetized and immobilized in a stereotactic apparatus. Next, after the induction of general anesthesia, a particular type of suspension was transcranially implanted/administered to their left brain hemispheres (coordinates antero-posterior: −0.30 mm; medio-lateral: 3.0 mm; dorso-ventral: 5.0 mm [67]). The initial stage was to drill a hole in the rat’s skull, into which a needle (27-gauge needle on a Hamilton syringe) with the cell suspension was then placed. One minute after the insertion of the needle, a 5-microliter volume of cell suspension was injected into the brain, and the needle was slowly removed after 3 min. The wound was then stitched with a stapler and sanitized. Animals were awakened from anesthesia a few minutes after completing the implantation procedure. The rats subjected to the implantation of glioma cells were, afterwards, daily immunosuppressed with cyclosporine A (Novartis Poland at a daily dose of 5 mg/kg of body mass) and observed in terms of behavioral changes. In preliminary studies, taking into account the health status of the animals after the implantation (weight loss, neurological problems), the survival time was determined in the respective groups, 21 days for the Pa, T and M groups and 15 days for the U group. At the end of the experiment, the rats received a euthanasia agent (Euthasol vet. 400 mg/mL, Le Vet) and, afterwards, they were subjected to perfusion with a physiological saline of high analytical purity. The brains removed from the skulls were frozen in liquid nitrogen and subsequently cut into 20-micrometer-thick slices using cryomicrotome. Tissue sections taken from the place of implantation were used for further analysis. These specimens were mounted on the Ultralene foil and freeze-dried.

For the qualitative and quantitative elemental analysis of brain slices, the SR-XRF microscopy was applied. The measurements were performed at the FLUO beamline at the KIT Synchrotron Light Source in Karlsruhe, Germany [68]. The X-ray beam energy of 16 keV enabled efficient detection of the following elements: P, S, K, Ca, Fe, Cu, Zn and Se. A beam size of 200 μm × 200 μm was enough to differentiate the main brain structures as well as the area of tumor/implantation. Typically, the acquisition time of a single XRF spectrum was 5 s per pixel.

## 5. Conclusions

The local elemental anomalies observed in our study were correlated with the histological changes of brain tissue that resulted from tumor expansion. The tumors developing from U87mg cells and patient-derived cells were characterized with the increased accumulation of Fe and Se, whilst the tissue directly surrounding the tumor presented a higher areal density of Cu. The tissues taken from animals subjected to the implantation of T98g cells were similar to those taken from the rats that obtained the culture medium, in terms of both morphology and the elemental distribution. Although no signs of tumor development were found for these groups, the tissue damage connected with the administration of cells or culture medium was observed and characterized by an increased accumulation of Fe and Ca.

## Figures and Tables

**Table 1:** Detection limits of analyzed elements with uncertainties calculated as standard deviation of the mean value.

Element	P	S	K	Ca	Fe	Cu	Zn	Se
LOD (ng/cm^2^)	355.4	122.7	39.36	12.25	2.695	1.126	1.544	0.4493
SD (ng/cm^2^)	3.8	1.3	0.41	0.13	0.028	0.012	0.015	0.0073

**Table 2:** Characteristics of examined animal groups.

Animal Group	Characteristics
N	Naive, normal rats (without implantation)
M	Implantation of 5 μL of Dulbecco’s Modified Eagle Medium (DMEM) used for preparation of cell suspensions
Pa	Implantation of human GBM cells extracted from a patient, suspended in 5 μL of DMEM, 50,000 cells/µl
T	Implantation of cultured human GBM cell line T98g, suspended in 5 μL of DMEM, 50,000 cells/µl
U	Implantation of cultured human GBM cell line U87mg, suspended in 5 μL of DMEM, 5000 cells/µl

## Data Availability

The data that support the findings of this study will be made available after reasonable request. We may require the participation in the authorship after the use of the shared data.
